# Ontogeny, evolution and palaeogeographic distribution of the world’s largest ammonite *Parapuzosia* (*P*.) *seppenradensis* (Landois, 1895)

**DOI:** 10.1371/journal.pone.0258510

**Published:** 2021-11-10

**Authors:** Christina Ifrim, Wolfgang Stinnesbeck, Arturo H. González González, Nils Schorndorf, Andrew S. Gale

**Affiliations:** 1 Institut für Geowissenschaften, Ruprecht-Karls-Universität Heidelberg, Heidelberg, Germany; 2 Museo del Desierto, Saltillo, Coahuila, Mexico; 3 School of the Environment, Geography and Geological Sciences, University of Portsmouth, Portsmouth, United Kingdom; 4 Earth Sciences Department, Natural History Museum, London, United Kingdom; University of California, UNITED STATES

## Abstract

The world’s largest ammonite, *Parapuzosia* (*P*.) *seppenradensis* (Landois, 1895), fascinated the world ever since the discovery, in 1895, of a specimen of 1.74 metres (m) diameter near Seppenrade in Westfalia, Germany, but subsequent findings of the taxon are exceedingly rare and its systematic position remains enigmatic. Here we revise the historical specimens and document abundant new material from England and Mexico. Our study comprises 154 specimens of large (< 1 m diameter) to giant (> 1m diameter) *Parapuzosia* from the Santonian and lower Campanian, mostly with stratigraphic information. High-resolution integrated stratigraphy allows for precise cross-Atlantic correlation of the occurrences. Our specimens were analysed regarding morphometry, growth stages and stratigraphic occurrence wherever possible. Our analysis provides insight into the ontogeny of *Parapuzosia* (*P*.) *seppenradensis* and into the evolution of this species from its potential ancestor *P*. (*P*.) *leptophylla* Sharpe, 1857. The latter grew to shell diameters of about 1 m and was restricted to Europe in the early Santonian, but it reached the Gulf of Mexico during the late Santonian. *P*. (*P*.) *seppenradensis* first appears in the uppermost Santonian- earliest Campanian on both sides of the Atlantic. Initially, it also reached diameters of about 1 m, but gradual evolutionary increase in size is seen in the middle early Campanian to diameters of 1.5 to 1.8 m. *P*. (*P*.) *seppenradensis* is characterized by five ontogenetic growth stages and by size dimorphism. We therefore here include the many historic species names used in the past to describe the morphological and size variability of the taxon. The concentration of adult shells in small geographic areas and scarcity of *Parapuzosia* in nearby coeval outcrop regions may point to a monocyclic, possibly even semelparous reproduction strategy in this giant cephalopod. Its gigantism exceeds a general trend of size increase in late Cretaceous cephalopods. Whether the coeval increase in size of mosasaurs, the top predators in Cretaceous seas, caused ecological pressure on *Parapuzosia* towards larger diameters remains unclear.

## Introduction

**Fig 1:**
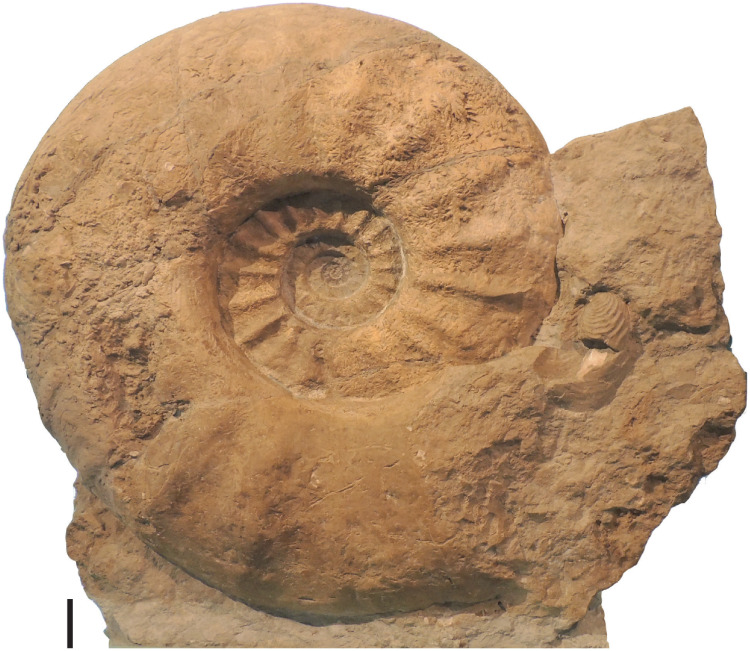
Lectotype of *Parapuzosia* (*P*.) *seppenradensis* from Westfalia, Germany. The specimen was collected in 1894 near Seppenrade. LWL-Museum for Natural History, Münster. Photo: CI. Scale: 100 mm.

The lectotype of the world’s largest ammonite, *Parapuzosia (P*.*) seppenradensis* (Fig 1), is exhibited at the entrance of the Museum of Natural History (Landesnaturkundemuseum) in Münster (LWL), but replicas of this original reaching 1.74 m diameter are displayed in many museums worldwide. The specimen has not been compared to other *Parapuzosia* species due to the scarcity of specimens with comparable size [1–3]. 154 specimens from the UK, France, Germany, North America are included for the present review, among them all historic specimens from Europe and the US and abundant new material from England and Mexico. Stratigraphic information is available for most of these specimens. Diameters range from 0.1 m to 1.80 m, thus allowing us to study the complete ontogeny of *Parapuzosia (P*.*) seppenradensis* up to its maximum size.

## Methods

For this study, all available specimens of *Parapuzosia (P*.*) leptophylla and P*. *(P*.*) seppenradensis* were recorded with morphometry and photodocumentation. At Tepeyac, the stratigraphic position of specimens in the section was registered as bed number, and ages were assigned based on associated ammonoids, inoceramids, and the stable carbon isotope curve. At Peacehaven, Sussex, UK, 40 specimens were measured on the foreshore of the beach. About 60 additional specimens were located there, but these were not suitable for morphometric measurements due to insufficient preservation. Their stratigraphic position was registered as height above or below lithostratigraphic marker beds, e.g. the Old Nore Marl. In both localities, the position of each specimen was measured by GPS. A.kmz file is available from the corresponding author upon request.

Morphometry was taken at various diameters wherever possible (whorl width, whorl height, diameter of umbilicus, number of ribs per whorl including secondaries), in addition to the ontogenetic stage and stratigraphic level and age. Diameters in morphometry refer to location of measurement on the individual, of histograms to size of the phragmocone, i.e. the diameter at the last suture. Preserved diameter refers to the maximum diameter visible in a specimen, although in most shells the original diameter was larger but is not preserved due to broken apertures. Rib index refers to both primary and secondary ribs, counted from the given diameter downwards. Where necessary the number of ribs per whorl was estimated by the multiplication of rib numbers per half whorl with the factor 1.9, resulting from observation on complete whorls. The calculated diameter of a specimen includes the body chamber which is rarely preserved; it was calculated by multiplying the diameter at the last suture, i.e. of the phragmocone, by a factor of 1.5. This factor was estimated based on complete Tepeyac specimens which preserve the maximum angle of body chamber (220°); it refers to the relation total shell diameter D/phragmocone diameter DP. The original diameter of specimen PH1 from the Littlehampton Museum was estimated based on the observation that the succeeding whorl was fully septate, as indicated by remains of shell and septae of this succeeding whorl up to the preserved end of the phragmocone.

Correlation of the English Chalk sections with the Mexican Tepeyac section is based on the stable carbon isotope curve published by [4] from Seaford Head as well as own data. 109 samples for stable carbon- and oxygen-isotope analyses on bulk carbonates were taken over the Tepeyac section at a resolution of 0.25 m (where possible). Bulk samples were dried, ground, and analyzed using a ThermoFisher MAT253plus with Kiel IV carbonate preparation at the Institute of Geosciences of the Ruprecht-Karls-Universität Heidelberg. All isotope values are reported in ‰ relative to the Vienna Peedee belemnite standard. The East Kent and Sussex sections are correlated by bio- and lithostratigraphy. Correlation of the Tepeyac succession with the lithostratigraphy of the Münsterland, Germany, is based on the position of the Santonian-Campanian boundary and on ammonoid zonation, particularly the FO of *Scaphites hippocrepis* III.

Palaeogeographic distribution through time was carried out by estimation of the numerical age of the occurrences from correlation with the Geological Timescale 2016 [5].

## Results

### Giant *Parapuzosia* species

In our material, we identified two species of *Parapuzosia* with shell diameters of >1 m. One is *P*. (*P*.) *leptophylla* Sharpe, 1857 [6], which was recently re-described formally [7]. 11 of the 154 specimens studied here were assigned to this species; they were originally collected from the Santonian of England, France, Germany and Mexico. The other giant species is *P*.(*P*.) *seppenradensis* (Landois, 1895) [1] which grew to 1.50 m shell diameter and even to ca 1.80 m as seen in the lectotype. An emended diagnosis for *P*.(*P*.) *seppenradensis* (Landois, 1895) is here presented in S1 File. 143 specimens from the uppermost Santonian and lower Campanian of Germany, England, Mexico and the USA are here included into *P*. (*P*.) *seppenradensis*.

**Fig 2:**
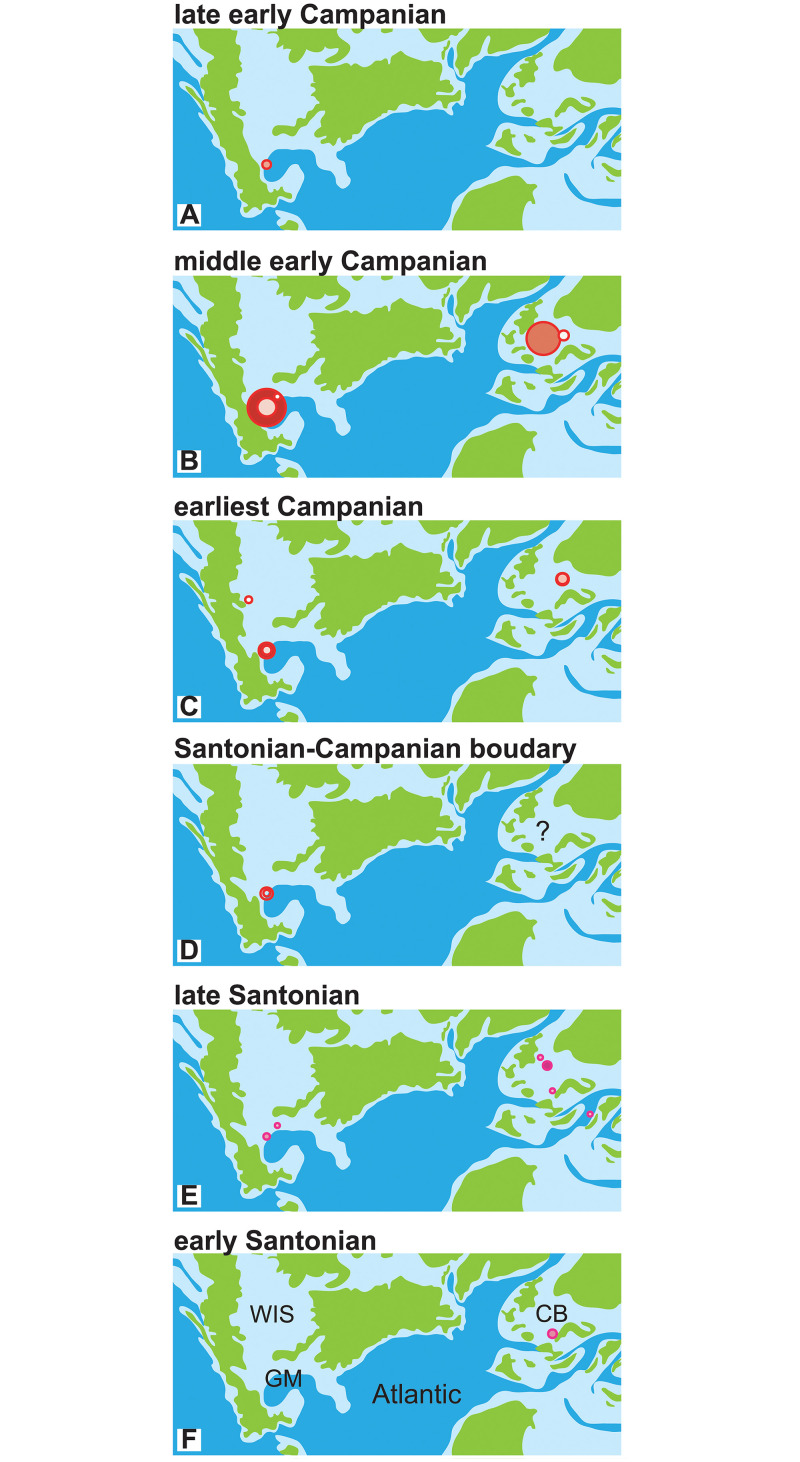
Distribution of giant *Parapuzosia* in the Santonian—Early Campanian. Area of dots approximates abundance of specimens. Abbreviations: CB: Cretaceous Basins of Europe, GM: Gulf of Mexico, WIS: Western Interior Seaway. Maps modified [from 8] under a CC BY license, with permission from deeptimemaps.org, original copyright 2016.

Geographic occurrences of large and giant *Parapuzosia* are summarized in Fig 2. The specimens, their origin, age, morphometry and further details are summarized in S1 Table.

### Occurrence

#### European continental occurrences

Ten specimens of *P*. (*P*.) *seppenradensis* are today known from the lower Campanian of Westfalia, Germany [2]. Three of these specimens, including the lectotype, are housed at the LWL in Münster [3], while a fourth individual is mounted in a pub in Seppenrade [2], the village from which three specimens originated. They were found in sandstone assigned to the Dülmen Formation. Six smaller-sized (0.275 to 0.78 m) specimens of *P*. (*P*.) *seppenradensis* found in sandy marlstone north, west and southwest of Seppenrade, are housed in the Ruhrlandmuseum in Essen and in the Quadrat-Bottrop Museum for Pre- and Local History (Ur- und Ortsgeschichte) in Bochum, Germany [9,10].

A specimen of ca. 1 m diameter from the late Santonian of the Gosau region of Austria was originally assigned to *P*. (*P*.) cf. *seppenradensis* [11], but was later included into *P*. (*P*.) cf. *leptophylla* [7], an interpretation followed here.

Two specimens of *Parapuzosia* (*P*.) *leptophylla* have been recorded from lower Santonian sediments of southwestern and southeastern France, the latter occurring in shallow marine facies [12].

#### *Parapuzosia* from England

**Fig 3:**
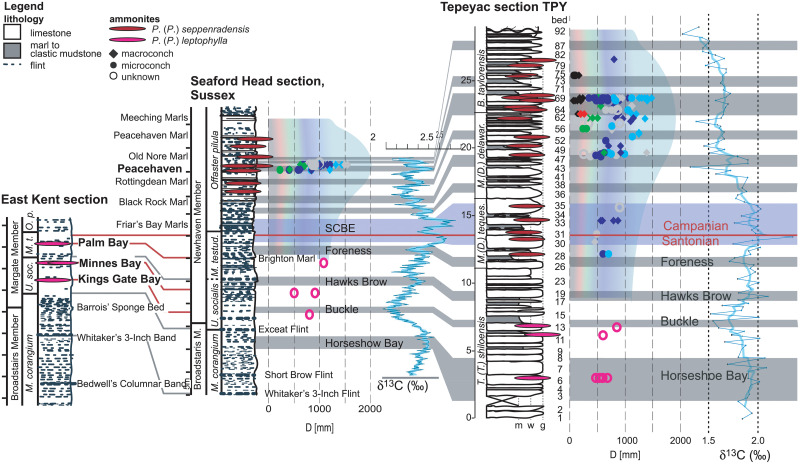
Stratigraphic distribution of *Parapuzosia* and their diameters from the English Chalk of east Kent and Sussex, U.K., and from Tepeyac, northern Coahuila, Mexico. Chalk sections are [redrawn from 14, 15]. Colors refer to growth stages in *P*. (*P*.) *seppenradensis* (see also Fig 5). δ^13^C distribution in the Chalk from [4]. The East Kent and Sussex sections are correlated by bio- and lithostratigraphy, correlation to Mexico by δ^13^C chemostratigraphy. Abreviations for biostratigraphy: *M*. *corangium*: *Micraster corangium M*. (*D*.) *delaw*.: *Menabites* (*Delawarella*) *delawarense*, *M*. (*D*.) *tequesqu*.: *Menabites* (*Delawarella*) *tequesquitense*, *M*.*t*. and *M*. *testud*.: *Marsupites testudinarius*, *O*. *p*.: *Offaster pilula*, *U*. *soc*.: *Uintacrinus socialis*.

Specimens of giant *Parapuzosia* in the English Chalk in Kent and Sussex, UK, were first mentioned by Sharpe [6] and Spath [13], but few have been collected. We located *P*. *(P*.*) leptophylla* in upper Santonian chalk cliffs in east Kent, at Minnis Bay (two), Palm Bay (one), Kingsgate Bay (one), and *P*. *(P*.*) seppenradensis* in foreshore outcrops of lower Campanian chalk at Peacehaven in Sussex (ca. 100). The stratigraphic distribution of these specimens within the English Chalk is shown in Fig 3. The inner whorls, measured to 0.53 m, of a large Peacehaven specimen estimated to 1.50 m shell diameter, are housed in the local museum of Littlehampton, Sussex.

#### Historic specimens from North America

Giant specimens of *Parapuzosia* are also known from the US. A specimen of 1.03 m diameter from SE Texas was described as *Parapuzosia americana* Scott and Moore, 1928, while a second individual of 0.95 m diameter was assigned to *Parapuzosia bosei* Scott and Moore, 1928. Both are from micritic limestone of the Austin Group and were collected at about 40 km north of Eagle Pass, on the Del Rio road along the Rio Grande river [16]. Adkins [17] briefly refers to these specimens and the locality; Böse [18] discovered localities with large “*Pachydiscus”* in Texas and Mexico but died before he could work out details. His material was described by Renz [19] and, among other ammonites, by Young [20]. A specimen, 1.25 m in diameter and described as *Parapuzosia bradyi* n.sp., was documented by Miller and Youngquist [21] from the Eagle Ford Shale of Montana. None of these historical North American specimens has yet been assigned to a precise stratigraphical level within the Santonian-early Campanian interval. Kennedy [in 22] documented a small (0.18 m) specimen assigned to *P*. *bosei* from the uppermost Santonian of central Texas.

CI and WS revised 11 well preserved specimens of giant *Parapuzosia* housed in official (museum, university, public agencies) collections in the Mexican states of Chihuahua, Coahuila and Nuevo León. They are all preserved as internal moulds in micritic limestone, and all specimens originate from the Jiménez area in the northeast of the Mexican state Coahuila (see Suppl. 2 for location and geology).

#### *Parapuzosia* from Tepeyac, Mexico

The site studied here is located near the village of Tepeyac, about 40 km north of Piedras Negras and 15 km SSW of Jiménez, Coahuila (28° 54’ 45’’N, 100° 44’ 29’’W). It is situated in the bed of the intermittent Arroyo Blanco river, a tributary to the Rio Grande, and close to the mouth of the Arroyo Tecolote (S4 Fig). The locality likely corresponds to the original site of Böse [18], Renz [19] and Young [20] (S2 File).

The Arroyo Blanco and Arroyo Tecolote riverbeds expose the same beds. We logged a section of 28.9 m conformed by rhythmically bedded bright grey to light yellow-coloured layers of chalk and limestone [interpreted as wacke- and packstone sensu 23], and intercalated units of marl and marly clay. The beds dip by about 3° to the east and are well exposed over tens to hundreds of meters along the dry river bed, allowing for a detailed search for fossils over hundreds of square meters on the top of numerous layers up to bed 69. Beds 70 to 92 are exposed along the riverbank, and they are very fossiliferous. We discovered a total of 66 *Parapuzosia* specimens, (14 internal and 52 external moulds), in addition to about 500 other mollusks. Based on our biostratigraphic zonation and stable carbon isotopic analysis, summarized in Fig 3, the base of the section is assigned to the lower *Texanites shiloensis* Zone of the upper Santonian and the top dated to the *Baculites taylorensis* zone of the upper lower Campanian [see e.g. 24].

*Parapuzosia* specimens are embedded laterally or slightly obliquely. Recrystallized shell material was identified in two individuals. In addition to moulds, imprints are also abundant and most are sufficiently well preserved to provide details for morphometric analysis. Most of the Tepeyac *Parapuzosia* show damage to the body chamber, including broken apertures, or the body chamber is completely lost. Aptychi are preserved in the body chambers of specimens CPC–1001 of unknown origin, in CPC–2204 collected from bed 74, and in P8 (not collected) from bed 33 of the Tepeyac section. Even when the body chamber is preserved, it is difficult to extract.

*Parapuzosia* reaching from 0.10 to 1.48 m preserved diameter were identified by us throughout the upper Santonian to lower Campanian Tepeyac section. Broken apertures in these specimens may indicate that they were transported *post-mortem*. In other individuals, undamaged apertures and the presence of aptychi in the body chamber indicate that *post-mortem* transport distance was minor.

Specimens collected from beds 1–69 of the Tepeyac section are grey-coloured, while those from beds 70–92 are yellow. An outcrop of coeval sediments at Arroyo Fresno at about 10 km north of the Tepeyac section yielded giant *Parapuzosia* preserved in yellow limestone; these specimens (e.g. CPC–1000) are here tentatively correlated to the middle-upper portion of the Tepeyac section.

## Ontogeny, stratigraphic and palaeogeographic distribution of *Parapuzosia* (*P*.) *leptophylla* and *P*. (*P*.) *seppenradensis*

### Ontogeny and dimorphism

Phragmocone diameters of the Tepeyac *Parapuzosia* range from 0.10 to 1.65 m. Based on the last suture preserved, the original size of the largest specimen can therefore be calculated to about 2 m. This specimen (TPY P19, S2K Fig) thus represents the largest ammonite of North America and the second largest in the world. The enormous size range identified in the 143 specimens revised here allows for a discussion of individual growth and the differentiation of five ontogenetic stages in *P*.(*P*.) *seppenradensis*, but also a concise differentiation between this species and *P*. *(P*.*) leptophylla*.

**Fig 4:**
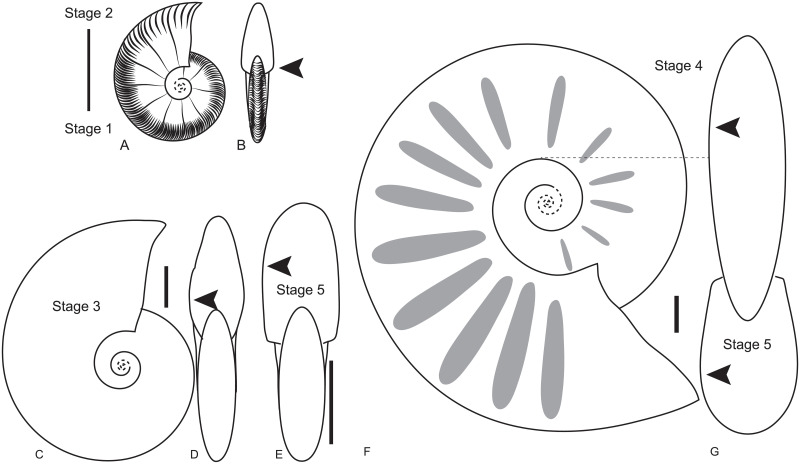
Ontogenetic stages of *Parapuzosia* (*P*.) *seppenradensis* from the Jiménez area of Coahuila, north-eastern Mexico. A–B: juvenile stage 1, drawn from CPC–2204 from bed TPY 77. C–D: juvenile stage 3 (inner) and 4 (outer whorl), drawn from CPC–2206, macroconch from bed TPY 57 in lateral (C) and apertural view (D). E: juvenile stages 3–4 (inner) and 5 (outer whorl), drawn from CPC–2201, microconch from bed TPY57. Note that stage 5 is reached at a rather small diameter. F-G: macroconch juvenile stage 4, subadult stage 5 in the last quarter whorl, drawn from CPC–1000, precise origin within the Jiménez area is unknown. Drawings by CI. Scale bar = 100 mm.

The smallest individuals of *P*.(*P*.) *seppenradensis*, with phragmocone diameters of up to 0.11 m, show about 9 sharp primary ribs per half whorl, intercalated by seven secondary ribs, with their number gradually decreasing to three with increasing diameter (D). We refer to the morphology of these small-sized specimens as juvenile stages 1 (seven secondaries, Fig 4E and 4F) and 2 (three secondaries e.g. [22]). Secondaries disappear between 0.20 m and 0.40 m diameter, and subsequent ornamentation consists solely of straight primary ribs, or shells are smooth (juvenile stage 3, S2A and S2B Fig, Fig 4C and 4D). The shell is approximately discoidal in juvenile stage 3 and has convergent flanks with initially thin, sharp and very regular ribs; this stage is followed by broader and wider-spaced ribs, here termed juvenile stage 4, providing greater whorl width with convergent flanks, but still a narrowly rounded venter (S2A, S2B and S2C–S2G Fig). This gradual transition is identified at phragmocone diameters ranging from 0.30 to 0.60 m. Larger shells show a gradual transition of convergent to parallel flanks, while the venter changes from narrowly to widely rounded (growth stage 5, Figs 1, 4F and 4G, S2J Fig). This is the last growth stage, which is here interpreted to represent the adult ontogenetic stage. The transition from growth stage 4 to growth stage 5 is gradual and completed at differing diameters ranging from 0.50 m to 1.40 m. At diameters between 1.0 and 1.5 m, ribs become even wider, more undulating and irregular, leading to an almost smooth lateral surface at largest diameters (>1.5 m, Figs 1, 4F and 4G, S2J and S2K Fig). The growth stage is also characterized by the position of greatest whorl breadth (arrows in Fig 4). It is situated near the umbilicus in growth stage 1 and 2, on the inner flank in stage 3, and approximating a mid-flank position in growth stage 4; the greatest whorl breath is situated at ca. 1/3 whorl height on the outer half in growth stage 5.

The lectotype of *P*. (*P*.) *seppenradensis* has parallel flanks, a broadly rounded venter, and wide, shallow, regular ribs throughout the last whorl, i.e. growth stage 5. The paralectotype of *P*. *seppenradensis*, as well as specimen WMN 5159 from Dülmen and a fourth individual from Seppenrade, also agree with ontogenetic stage 5 as established based on the Tepeyac material. The Tepeyac specimens of *Parapuzosia* are therefore assigned to *P*. (*P*.) *seppenradensis* Landois, 1895.

Most specimens from the Jiménez (Tepeyac) region and adjacent Texas, originally assigned to various species of *Parapuzosia*, fall into growth stage 3 (see S1 File). The holotype of *P*. *americana* Scott and Moore, 1928 [16] and the visible inner whorl of the holotype of *P*. (*P*.) *bradyi* Miller and Youngquist, 1946 [21] correspond to growth stage 4. Growth stage 5 corresponds to the morphotype of *P*. *bosei* Scott and Moore [16], 1928. These species are thus junior synonyms of *P*. (*P*.) *seppenradensis*.

The Santonian species *P*. (*P*.) *leptophylla* is distinguished from *P*. (*P*.) *seppenradensis* by irregular ribbing in growth stage 3 and by slightly flexuous ribs (S2E–S2G Fig). It is interpreted as adult in the smooth growth stage (stage 4) with convergent flanks, because this is the last growth stage observed. *P*. (*P*.) *leptophylla* never shows a rounded venter or parallel flanks near the aperture, although it is indistinguishable by standard morphometry alone (S3 Fig). The earliest growth stages of *P*. (*P*.) *leptophylla* are yet unknown.

**Fig 5:**
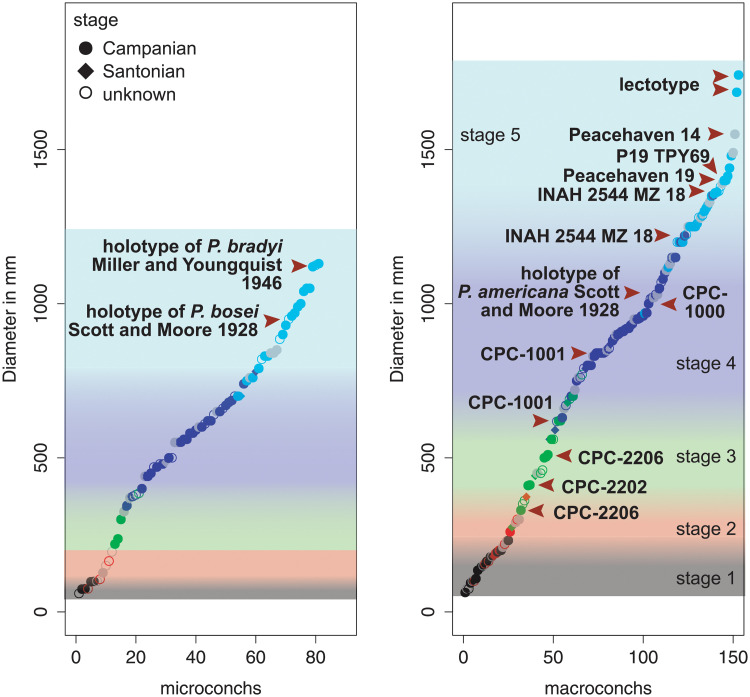
Shell diameter and associated growth stage in *Parapuzosia* (*P*.) *seppenradensis*. The specimens from Mexico, Texas, Montana, England and Germany are separated after microconchs (left) and macroconchs (right). Colours refer to ontogenetic stages.

The five ontogenetic growth stages described above for *Parapuzosia (P*.*) seppenradensis* are reached at varying shell diameters, shown e.g. in Fig 3. The specimens can be divided into two coeval groups of individuals (Fig 5), which is here explained by size dimorphism. The effects of dimorphism overlap with the evolutionary increase in shell size (see 3.2). This is especially seen in latest Santonian morphotypes which cover the lower range of diameters of transition between growth stages. In Campanian specimens, however, both maximum sizes and widest shell size variations are identified in both North American and European specimens. Dimorphism was earlier suggested for *Parapuzosia (P*.*) seppenradensis* [2], but based on a single specimen which is here referred to ontogenetic stage 4 and thus considered to be a juvenile macroconch.

### Size

Diameters of *Parapuzosia* specimens generally increase throughout the Tepeyac section reaching maxima in bed 69 (Fig 3, S4 Fig). While *P*. (*P*.) *leptophylla* reaches shell diameters of 0.50 to 0.70 m in the upper Santonian part of the Tepeyac section (beds TPY 1–20), specimens of *P*. *(P*.*) seppenradensis* collected from stratigraphically younger layers (beds TPY 21 to 40) reach adult sizes of about 1.20 m. Largest shell sizes of about 1.50 m (or 2.0 m including the body chamber) are present in the lower Campanian section (beds TPY 50–69); in these individuals, adult (stage 5) morphologies are first seen at shell diameters of ca. 0.75 to 1.30 m. The shell size of *Parapuzosia*, but also diameters of transition from one growth stage into the next, thus increases across the Santonian- Campanian boundary in both the Gulf of Mexico and the English Chalk, with largest diameters reached in the middle early Campanian on both sides of the Atlantic (S4 Fig). This stratigraphic level is approximately coeval to the layer in which the giant lectotype of *P*. *(P*.*) seppenradensis* was found in Germany, while smaller specimens seem to originate from beds above and below [2]. Specimens from the uppermost lower Campanian are scarce and invariably of diameters < 1m.

### Stratigraphic and palaeogeographic distribution, and depositional environment

Palaeogeographic occurrences of giant *Parapuzosia* are shown in Fig 2. Early Santonian records of *P*. (*P*.) *leptophylla* exist from southern France [12]. In the late Santonian, the species is known from Austria, England [7] and Mexico, but it disappears prior to the Santonian-Campanian boundary, most possibly because it evolves into *P*. (*P*.) *seppenradensis*. The oldest records of this latter species are from the uppermost Santonian of Texas [22] and Mexico, its absence in Europe may be explained by a scarceness of outcrops. In the early Campanian, *P*. (*P*.) *seppenradensis* reaches its widest distribution, with records from Mexico, Texas, England, Germany, and possibly Montana. The species is thus distributed throughout the Central European Cretaceous basins and the northwestern Gulf of Mexico. It then becomes rare and smaller-sized in both Mexico (Tepeyac) and in England (Sussex).

The abundance of *Parapuzosia*, as expressed by the number of individuals per layer, increases towards the upper (lower Campanian) part of sections in both the English Chalk and Tepeyac (Mexico), unrelated to outcrop conditions. In both outcrop areas, coeval sediments elsewhere in the region are devoid of *Parapuzosia* shells. Near Ojinaga in northern Chihuahua, for instance, only about 300 km distance to the west of Tepeyac (S5 Fig), the expanded and exceedingly fossiliferous [25,26] Ojinaga Formation contains abundant *Placenticeras* and texanitids [27], but *P*. (*P*.) *leptophylla* and *P*. (*P*.) *seppenradensis* are absent. In southwestern Coahuila, lower Campanian sediments comprise a mostly endemic assemblage near Union y Progreso, including *Menabites* (*Delawarella*) *vanuxemi* and *Pseudoschloenbachia* (*P*.) *mexicana*, but without *Parapuzosia* [28]. The taxon is also characteristically absent along the Northern Gulf of Mexico and the Atlantic Coast of the US. A similarly patchy occurrence is seen in Europe. Abundant large specimens of *P*. (*P*.) *seppenradensis* are documented from Sussex in the southern UK and *P*. *(P*.*) leptophylla* has been found in the upper Santonian chalk of Kent. No reports exist from the early Campanian of France, but all German specimens of *P*. (*P*.) *seppenradensis* are from a small outcrop area in the Münsterland, a region of Westfalia.

This explicitly patchy geographic occurrence of *P*. (*P*.) *seppenradensis* appears to be unrelated to lithofacies. The taxon has been documented from coarse siliciclastic sediment in Montana [21] and the Münsterland [3], and from fine-grained carbonates in the case of the English Chalk and the Gulf of Mexico specimens. Possibly, the absence of *P*. (*P*.) *seppenradensis* in western Coahuila, in Chihuahua, and along the northern Gulf of Mexico coast line, may relate to the dominance of fine siliciclastic sediment in this region, but then its absence in the carbonate-dominated southern Chalk Basin of France remains to be explained.

Up to bed TPY69 of the Mexican Tepeyac section, *P*. (*P*.) *seppenradensis* is represented by subadults and adults. Above, in bed TPY70 and upsection, juvenile specimens dominate and larger shells are only occasionally present. The abundance of majorly adults followed by the dominance of early juveniles in the Mexican Tepeyac section suggests proximity to a mating and/or hatching area, which is shifted with sea-level changes. This would imply a monocyclic reproduction strategy for these giant ammonites [29,30].

Ammonoids have long been suspected to have employed a semelparous reproductive strategy, i.e. a spawning once strategy [e.g. 31–35]. Such a single spawning strategy is characterized by females producing thousands of eggs, typically laid in egg cases in mass spawning events. Adults die subsequently, although not necessarily immediately; in recent coleoids, mass accumulations of sexually mature adults are not usually found with their offspring. Accumulations of adult ammonoids have been interpreted as evidence of semelparous life cycles before [29]. A scenario of monocyclic reproduction with more than one spawning event comparable to recent coleoids [30] would also plausibly explain the abundance of giant *Parapuzosia* in a shallow shelf area and their absence in other nearby locations.

It should be noted that the Tepeyac and Peacehaven localities seem to comprise accumulation of subadults and adults. This indicates accumulation after a short reproduction phase. However, in the phase between hatching and spawning the individuals seem to have spent their life elsewhere, and most probably in deeper or even deep water. Records outside the Tepeyac and Peacehaven areas are isolated and unrelated to facies, they might represent isolated individuals of a species spending most of its lifetime offshore.

### Sea-level, temperature and climate

Two medium range 25–75 m eustatic sea-level lows (KSa2 and KSa3) have been identified during the late Santonian and one in the early Campanian (KCa1) [36], which likely caused intermittent decrease in water depth and expansion of shallow depositional environments in the western Gulf of Mexico. At Tepeyac, these regressive phases are reflected by the occurrence of trigoniids, alectrioniid oysters, and *Pinna* preserved in life position. The mid-early Campanian rise in sea-level, on the other hand, may be expressed by a change from white to yellow-coloured carbonates in the Tepeyac section, i.e. from shallow to substantially deeper shelf conditions. At about this lithological change, coincident with bed TPY69, largest specimens of *Parapuzosia* were detected and their abundance is highest. Above and below bed TPY69, *Parapuzosia* juveniles are abundant, and no adults were detected above bed TPY70. Upsection, *Parapuzosia* is absent in the topmost part of the Tepeyac section (to bed 92) dated to the upper lower Campanian (*Baculites taylorensis* Zone). In England, largest specimens are concentrated at a stratigraphic level approximately coeval with bed TPY69, and above this layer individuals also become increasingly rare. The size shift identified here in England and Mexico may thus have been coeval on both sides of the Atlantic. In extant cephalopods, the final body size often depends on hatchling sizes and on seasonal effects, e.g. water temperature during hatching and subsequent initial growth [37]. Hatching often occurs during a period of increasing water temperatures, i.e. in spring. In consequence, each monthly cohort encounters warmer temperatures and grows faster than cohorts that hatched weeks before [38]. Hatchling size is unknown for *Parapuzosia*. Within the Puzosiinae, hatchling size is only known for Turonian Pacific species [39] with narrow geographic and stratigraphic distribution and thus a low dispersal potential, different to *Parapuzosia* as interpreted here; they are thus not comparable. Other Desmoceratidae (Order: Ammonitina), into which *Parapuzosia* is also included, have hatchling size between 0.6 and 1.5 mm, most vary around 0.9 mm [39].

Even though, the importance of hatchling size may have been minor in giant organisms such as *Parapuzosia*. Instead, water temperature during growth may additionally have influenced final body and shell size, and may also explain the size variation within micro- and macroconchs. Nevertheless, it cannot explain the overall shift in size documented above for Campanian specimens.

The Cretaceous warm climatic conditions extended throughout the Santonian and into the Campanian, when a long-lasting cooling trend initiated during the early Campanian [40,41]. The δ^18^O curve [4] shows a minor drop at the Snt/Cmp boundary, followed by a phase of stability. During the Neogene, gradual oceanic cooling is interpreted to have resulted in a size increase of marine organisms, e.g. in whales [42]. The latest Cretaceous long-term cooling trend, initiating during the early Campanian, may thus have played a role for the size increase detected in *P*. *(P*.*) seppenradensis*. Nevertheless, this process started earlier. In addition, it would not explain the disappearance of this giant ammonite during the late early Campanian. The stable carbon isotope curve [4,15] shows a moderate peak across the Santonian-Campanian boundary, which does not correlate to an increase in shell diameter of *Parapuzosia* Fig 3); it thus seems to be unrelated. In consequence, no global trend is identified towards higher, or lower, temperatures, or changes in sea-level, that readily correlates with the increase in size and subsequent decrease of *P*. (*P*.) *seppenradensis*.

### Other large ammonoids and organisms in the Late Cretaceous seas

During the Late Cretaceous several genera of ammonoids developed large-sized species. These include the Cenomanian *Puzosia*, the Turonian *Pachydesmoceras* and *Lewesiceras*, the Santonian *Parapuzosia*, the early Campanian *Pachydiscus* (all in Central European Cretaceous basins), the late Campanian *Pachydiscus* (in the west Pacific) [43–45], and the early Maastrichtian *Pseudobaculites* (Western Interior Seaway) [46,47]. These giants thus represent different families and, in the case of *Pseudobaculites*, even orders. They were all recorded from regions which are relatively small in comparison with the distribution area of *P (P*.*) seppenradensis*. This latter species, though originating from one of these large forms, surpasses all others in both geographic distribution and size.

The gigantisms shown by the two species studied here may be a coevolution to the size increase in mosasaurs. These are known to have predated upon ammonoids [48] and considerably increase in size during the Santonian-early Campanian [49] parallel to *Parapuzosia*. However, the subsequent retrograde evolution of *Parapuzosia* from the late early Campanian on is clearly unrelated to the further increase in size of mosasaurs.

### Comparison with extant coleoids

Extant coleoids are the group most closely related to ammonoids [50]. These recent cephalopods are characterized by monocyclic spawning, although it is differentiated between a single synchronous ovulation (semelparity), several spawning events without ovary regeneration (with or without further growth between these events) or asynchronous ovulation with extended and continuous spawningPolycyclic spawning with regeneration of the ovary is restricted to *Nautilus* [30] which is more distantly related to ammonoids [50]. A monocyclic or even single-event spawning strategy is thus also plausible for *Parapuzosia* from an actualistic view.

*Architeuthis* is the largest existing coleoid. Based on genetics, this giant squid has been shown to be monospecific [*A*. *dux* (Steenstrup, 1857) [51]], even though it is characterized by high morphological variability [52] and global geographic distribution crossing latitudes [53]. In this respect it seems comparable to giant *Parapuzosia*. However, *Architeuthis dux* is even more widely distributed than *P*. *(P*.*) seppenradensis* was during the Campanian and seems to dwell and reproduce in deeper water depths than giant *Parapuzosia* did, although the water depth in which *Parapuzosia* dwelled during growth cannot be estimated from the known occurrences.

## Conclusions

The present analysis of giant *Parapuzosia* is based on 11 Santonian specimens of *P*. (*P*.) *leptophylla* and 143 latest Santonian to early Campanian individuals of *P*. (*P*.) *seppenradensis*, with preserved diameters of 0.1–1.8 m.

*P*. (*P*.) *leptophylla* was a rare species and majorly restricted to the Santonian of western Europe, reaching the western Gulf of Mexico during the late Santonian. The world’s largest ammonite, *P*. (*P*.) *seppenradensis*, appears to have evolved from this more widely distributed *P*. (*P*.) *leptophylla* in the very latest Santonian. *P*. *(P*.*) seppenradensis* is differentiated from its potential ancestor by an increase in body sizes, but also by minor morphological changes during ontogeny. All lower Campanian *Parapuzosia* are here assigned to *Parapuzosia* (*P*.) *seppenradensis*, including *P*. *americana* Scott and Moore, 1928 and *P*. *bosei* Scott and Moore, 1928, earlier described from Mexico and Texas, and *P*. *bradyi* Miller and Youngquist, 1960 from Montana, US. An increase in individual shell size is detected throughout the Santonian and into the early Campanian, where maximum shell sizes were reached on both sides of the North Atlantic. The species is thus widely distributed in shelf areas and epicontinental seas from North America to western Europe. Even though, the palaeogeographic distribution of *P*. (*P*.) *seppenradensis* is surprisingly patchy and its local geographic occurrence in North America and western Europe is difficult to explain, but may point to mating and hatching places and thus to a monocyclic and maybe even semelparous reproduction strategy, as repeatedly proposed for ammonoids. Size and abundance of *P*. *(P*.*) seppenradensis* decrease towards the end of the early Campanian. Whether the coeval increase in size of mosasaurs, the top predators in Cretaceous seas, caused ecological pressure on *Parapuzosia* towards larger diameters remains unclear. No other short-term global environmental trend is presently identified that correlates with the increase in size of this ammonite across the Santonian-Campanian boundary.

## Supporting information

S1 Fig Suture line of *P*. (*P*.) *seppenradensis*. Specimen CPC-1000 at D = 0.95 m.
(PDF)

S2 Fig Selected specimens of giant *Parapuzosia* from England (A-D) and northeastern Mexico (E-K). Some were used for Fig 2. A–B: *Parapuzosia* (*P*.) *seppenradensis*, specimen PH1 from Peacehaven beach, Littlehampton Museum, unregistered. C–D: *P*. (*P*.) *seppenradensis*, specimen PH19 on Peacehaven beach, not collected. E–G: *P*. (*P*.) *seppenradensis*, transitional to *P*. (*P*.) *leptophylla*, CPC–1001, ventral (E), lateral (F) and apertural (G) view, macroconch, fully septate, juvenile stage 4. H–I: *P*. (*P*.) *leptophylla*, CPC–2555 in lateral (H) and apertural (I) view. J: *P*. (*P*.) *seppenradensis*, largest collected ammonite of North America, macroconch, subadult stage 5, collection of Mauricio Fernandez Garza, DRPMZA—INAH 2544 MZ 18. K: specimen TPY-P19, largest ammonite of North America, not collected. Arrows point to the last suture. Scale: 100 mm.
(JPG)

S3 Fig Morphometry of latest Santonian and Campanian specimens of *Parapuzosia* (*P*.). Dimensions and relations are plotted against the shell diameter D (in mm). Colours refer to ontogenetic stages (see Fig 5). A: WB/WH, B: U/D, C: numbers of ribs per whorl, in part calculated by multiplicating the number of ribs per half whorl with factor 1.9.
(PDF)

S4 Fig Size classes of giant *Parapuzosia* (*P*.) over the Tepeyac and Chalk sections. Size refers to the diameter of the phragmocone (DP), i.e. diameter at the last suture; the indivdual shells were originally 1.5x larger. Magenta: *P*. (*P*.) *leptophlla*. Dark-red: *P*. (*P*.) *seppenradensis*. See Fig 3 for legend.
(PDF)

S5 Fig Map of Mexico with zoom on northern Coahuila. The Tepeyac section is marked by an asterisk in the geological map [modified from 73 under a CC BY license, original copyright 2008].
(PDF)

S6 Fig Lithofacies map [strongly modified from 74, 75 under a CC BY license with permission of the Servicio Geologico de Mexico, original copyright 1991] of northeastern Mexico and Texas. The Tepeyac locality is marked with a large asterisk. Red asterisks mark others with giant *Parapuzosia*, empty asterisks mark cephalopod occurrences coeval to Tepeyac in which giant *Parapuzosia* are absent. Fm: Formation.
(PDF)

S1 Table Database of the 154 specimens of *Parapuzosia* included into this study. (XLSX)

S1 File (DOCX)

S2 File (DOCX)
