# Supplementary figures and images for: Ontogeny, evolution and palaeogeographic distribution of the world’s largest ammonite Parapuzosia (P.) seppenradensis (Landois, 1895)
Source: PLoS One. 2021 Nov 10;16(11):e0258510. doi: 10.1371/journal.pone.0258510 (PMC8580234; doi:10.1371/journal.pone.0258510)

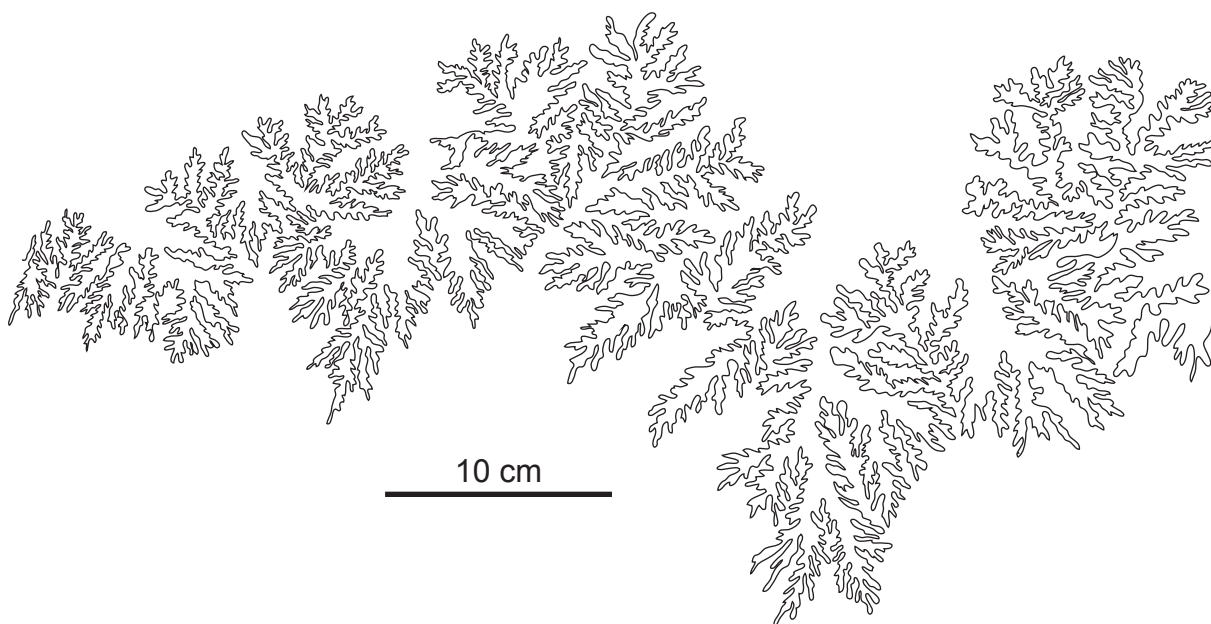

Supplement: S1 Fig — Specimen CPC-1000 at D = 0.95 m. (PDF) [file pone.0258510.s001.pdf]

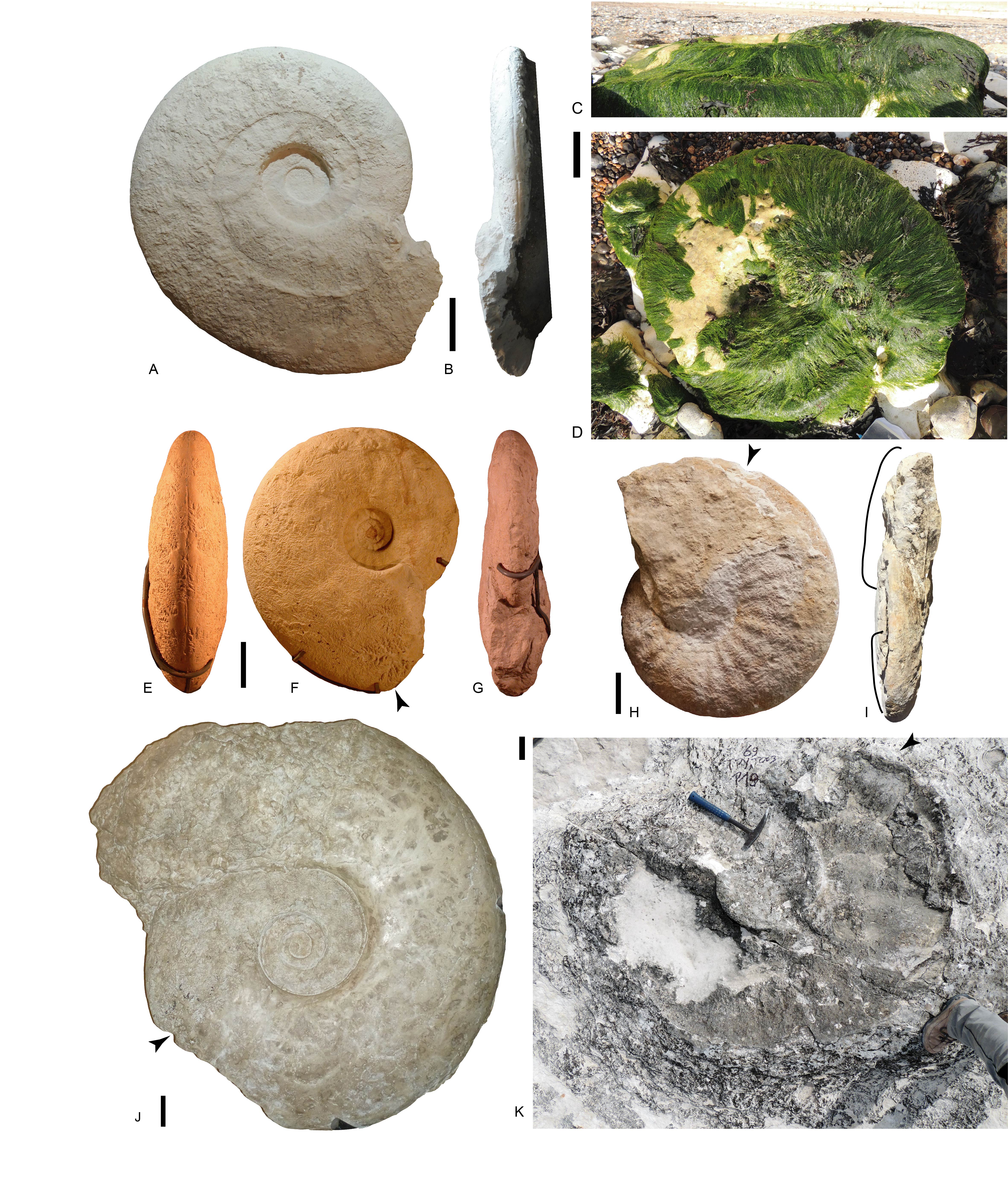

Supplement: S2 Fig — Some were used for Fig 2. A–B: Parapuzosia (P.) seppenradensis, specimen PH1 from Peacehaven beach, Littlehampton Museum, unregistered. C–D: P. (P.) seppenradensis, specimen PH19 on Peacehaven beach, not collected. E–G: P. (P.) seppenradensis, transitional to P. (P.) leptophylla, CPC–1001, ventral (E), lateral (F) and apertural (G) view, macroconch, fully septate, juvenile stage 4. H–I: P. (P.) leptophylla, CPC–2555 in lateral (H) and apertural (I) view. J: P. (P.) seppenradensis, largest collected ammonite of North America, macroconch, subadult stage 5, collection of Mauricio Fernandez Garza, DRPMZA—INAH 2544 MZ 18. K: specimen TPY-P19, largest ammonite of North America, not collected. Arrows point to the last suture. Scale: 100 mm. (JPG) [file pone.0258510.s002.jpg]

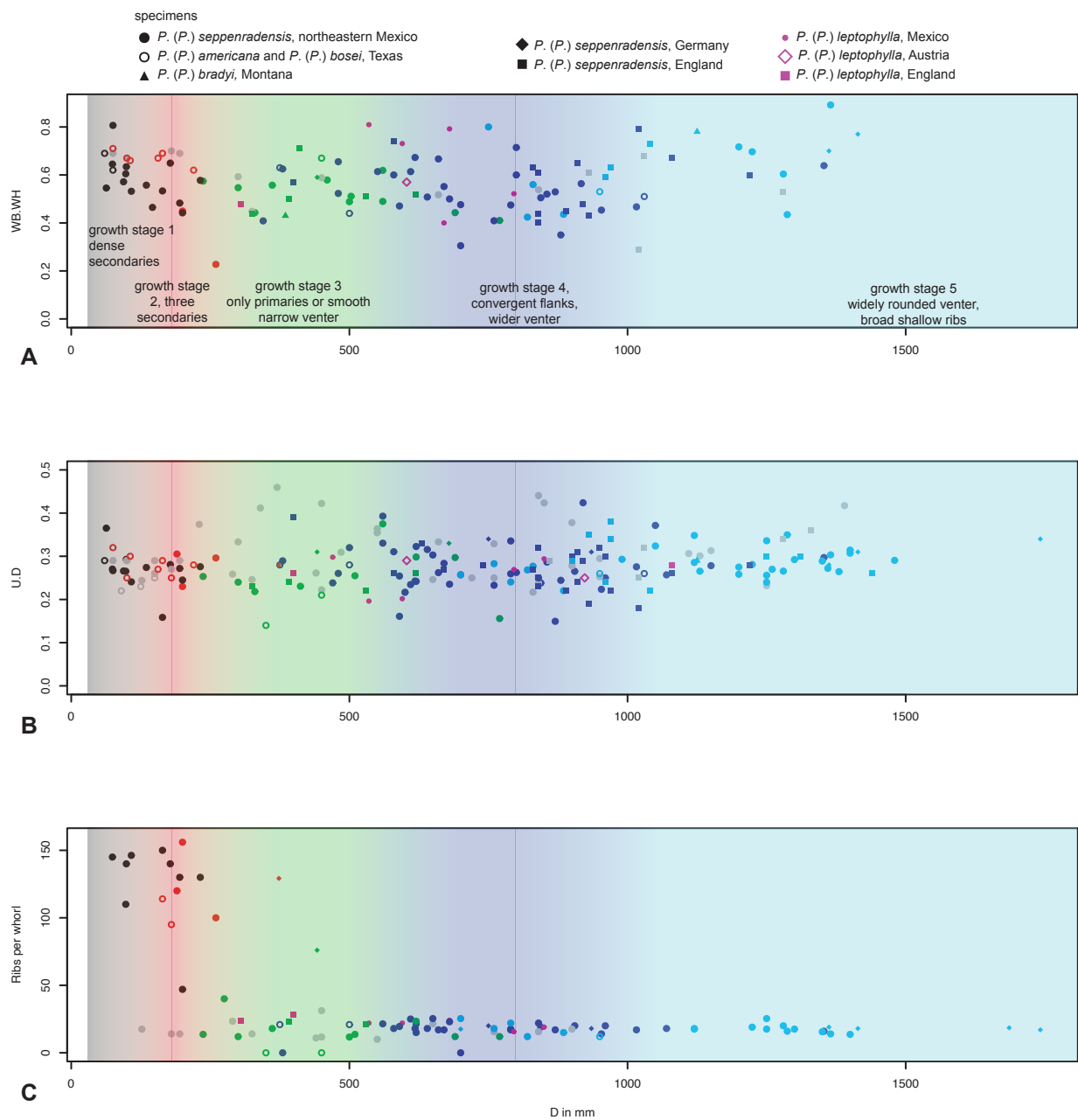

Supplement: S3 Fig — Dimensions and relations are plotted against the shell diameter D (in mm). Colours refer to ontogenetic stages (see Fig 5). A: WB/WH, B: U/D, C: numbers of ribs per whorl, in part calculated by multiplicating the number of ribs per half whorl with factor 1.9. (PDF) [file pone.0258510.s003.pdf]

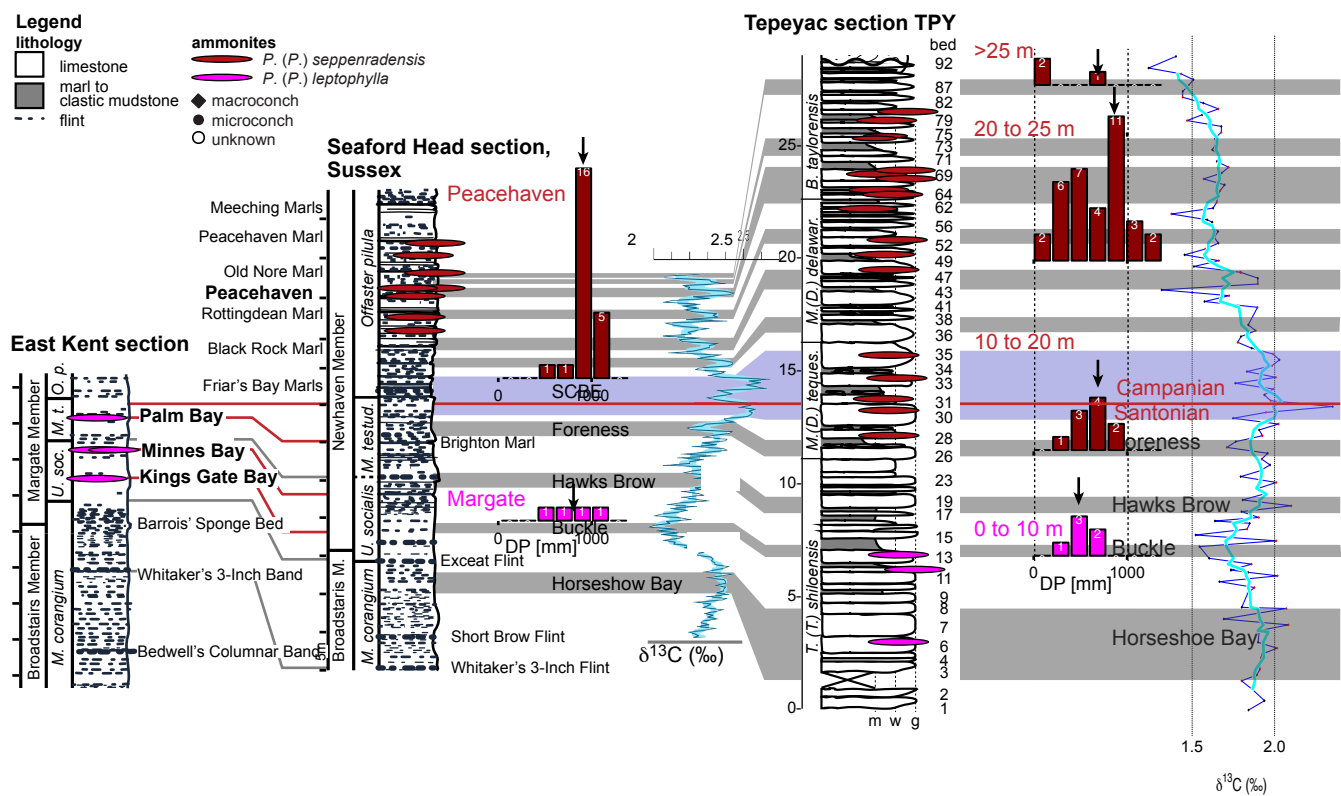

Supplement: S4 Fig — Size refers to the diameter of the phragmocone (DP), i.e. diameter at the last suture; the indivdual shells were originally 1.5x larger. Magenta: P. (P.) leptophlla. Dark-red: P. (P.) seppenradensis. See Fig 3 for legend. (PDF) [file pone.0258510.s004.pdf]

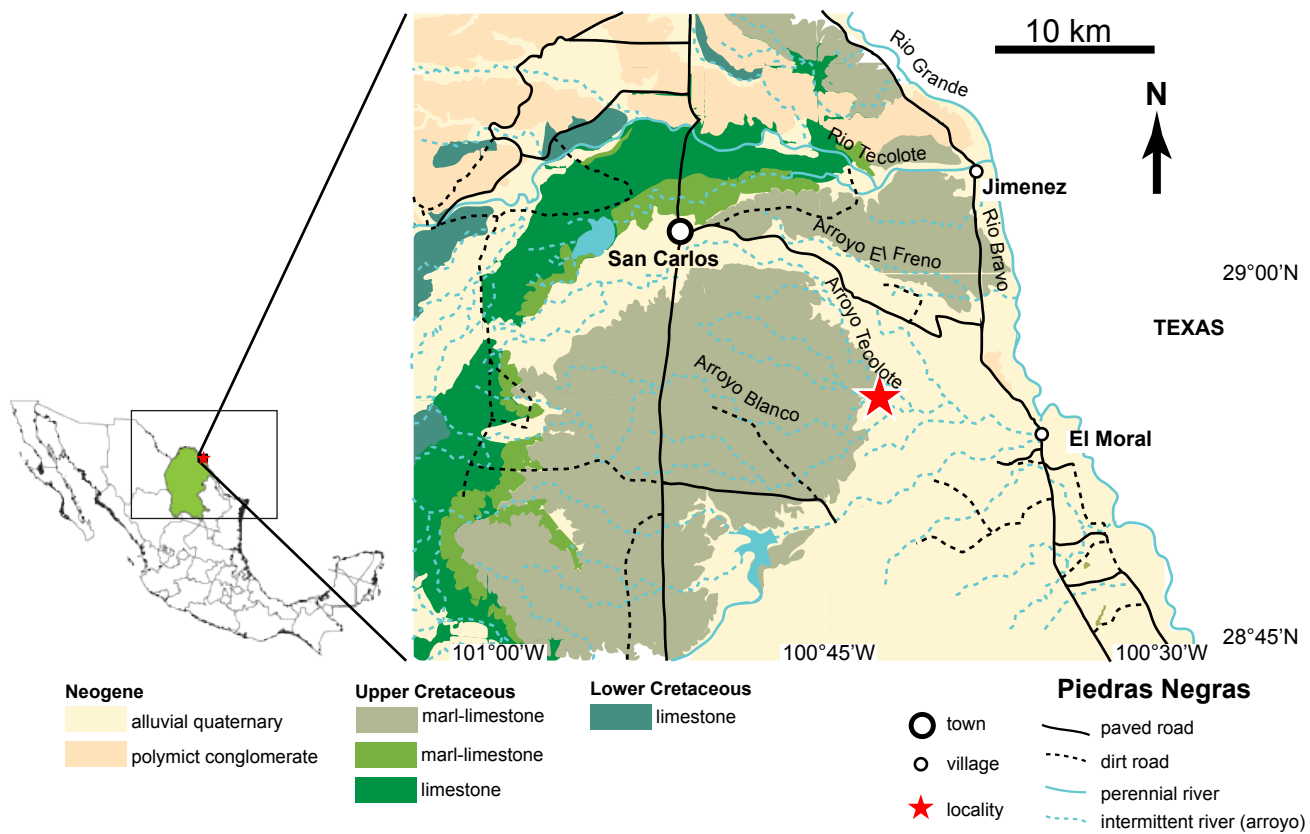

Supplement: S5 Fig — The Tepeyac section is marked by an asterisk in the geological map [modified from 73 under a CC BY license, original copyright 2008]. (PDF) [file pone.0258510.s005.pdf]
