# Supplementary material for: Ontogeny, evolution and palaeogeographic distribution of the world’s largest ammonite Parapuzosia (P.) seppenradensis (Landois, 1895)
Source: PLoS One. 2021 Nov 10;16(11):e0258510. doi: 10.1371/journal.pone.0258510 (PMC8580234; doi:10.1371/journal.pone.0258510)
Supplement: S6 Fig — The Tepeyac locality is marked with a large asterisk. Red asterisks mark others with giant Parapuzosia, empty asterisks mark cephalopod occurrences coeval to Tepeyac in which giant Parapuzosia are absent. Fm: Formation. (PDF) [file pone.0258510.s006.pdf]

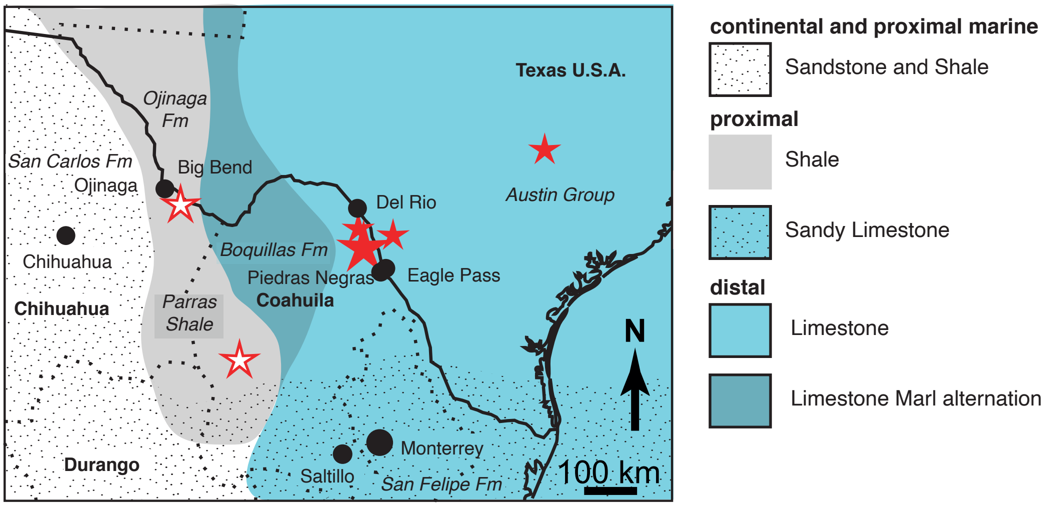
Texas U.S.A.
Ojinaga
Fm
San Carlos Fm
Big Bend
Ojinaga
Del Rio
Austin Group
Chihuahua
Boquillas Fm
Piedras Negras
Eagle Pass
Coahuila
Chihuahua
Parras
Shale
N
Monterrey
Durango
Saltillo
San Felipe Fm
100 km
continental and proximal marine
Sandstone and Shale
proximal
Shale
Sandy Limestone
distal
Limestone
Limestone Marl alternation
