# Supplementary material for: Ontogeny, evolution and palaeogeographic distribution of the world’s largest ammonite Parapuzosia (P.) seppenradensis (Landois, 1895)
Source: PLoS One. 2021 Nov 10;16(11):e0258510. doi: 10.1371/journal.pone.0258510 (PMC8580234; doi:10.1371/journal.pone.0258510)
Supplement: S1 File — (DOCX) [file pone.0258510.s008.docx]

# S1 File

Supplementary data

Ontogeny, evolution and palaeogeographic distribution of the world’s largest ammonite *Parapuzosia* (*P.*) *seppenradensis* (Landois, 1895)

Christina Ifrim*^1, 2^, Wolfgang Stinnesbeck^1^, Arturo H. González González^3^, Nils Schorndorf^1^, Andrew S. Gale^4^

# Systematic palaeontology

The systematics of ammonites follows the Treatise of Invertebrate Paleontology [54]. Dimensions are given in mm, dimensions in brackets are % of diameter. D: diameter; WH: whorl height; U: umbilical diameter. CPC: Colección de Paleontología de Coahuila, Museo del Desierto, Saltillo, Coahuila, Mexico.

#### Phylum Mollusca

#### Class Cephalopoda Cuvier, 1797 [1]

#### Order Ammonoidea Zittel, 1884 [2]

#### Suborder Ammonitina Hyatt, 1889[3]

#### Superfamily Desmoceratoidea von Zittel, 1895[4]

#### Family Desmoceratidae von Zittel, 1895[4]

#### Subfamily Puzosiinae Spath, 1922[5]

#### Genus and subgenus Parapuzosia Nowak, 1913

Type species: *Sonneratia Daubréei* de Grossouvre, 1894 (p. 154, pl. 28[54 ]) by original designation by Nowak (1913, p. 363).

##### Parapuzosia (Parapuzosia) seppenradensis (Landois, 1895)

Figs 1, 4A-G, S1, S2A-G, J-K, S3, S4, Table S1

1887 *Ammonites Coesfeldiensis* [sic] Schl.; Landois, p. 612-613. [7]

1891 *Ammonites coesfeldiensis* Schlüter; Fraas, p. 441. [8]

1895 *Pachydiscus seppenradensis* Landois, p. 1-10, pl. 1, 2. [9]

1899 *Ammonites* (*Pachydiscus*) *dülmenensis* Schlüter; Schlüter p. 414.[10]

1920 *Pachydiscus* *seppenradensis* Landois; Köplitz, p. 70-72, pl. 8, Fig 20.[11]

1927 *Pachydiscus flaccidicosta* Roemer; Böse and Cavins, p. 33. [12]

1928 *Parapuzosia bosei* Scott and Moore, 1928, p. 274, pl. 36, figs 1-3; pl. 37, Fig 2. (5) [13]

1928 *Parapuzosia americana* Scott and Moore, 1928, p. 275, pl. 37, figs 1, 3. ) [13]

1928 *Parapuzosia bosei* Scott and Moore, 1928; Adkins, p. 223. [14]

1928 *Parapuzosia americana* Scott and Moore, 1928; Adkins, p. 223. [14]

1928 *Pachydiscus flaccidicosta* Roemer; Böse, p. 172. [15]

1936 *Puzosia* (*Parapuzosia*) *corbarica* de Grossouvre; Renz, p. 5, pl. 4, figs 1–2. [16]

1946 *Parapuzosia bradyi* Miller and Youngquist, n.sp.; Miller and Youngquist, p. 481, pl. 37, figs 1-2, pl. 74, figs 1-2, pl. 75, figs 1-3. [17]

1963 *P. bosei* Scott and Moore, 1928; Young, p. 50, pl. 7, Fig 1; pl. 8, figs 1, 3-4; pl. 9, Fig 2; pl. 19, Fig 1; text-Fig 7j, q-r [18]

1963 *Parapuzosia* sp*.* aff. *P. bradyi* Miller and Youngquist, 1946; Young, p. 52, pl. 7, figs 2-3; pl. 9, figs 1, 3-4; pl. 11, Fig 1; text-Fig 8d. [18]

1963 *P. terryi* Young, p. 53, pl. 10, figs 2–4 [18]

*non* 1978 *Parapuzosia* cf. *seppenradensis* (Landois); Summesberger, p. 126, pl. 4, Fig 24; pl. 5, Fig 25; figs 14–15 (= *P.* (*P.*) *leptophylla*).[19]

*non* 1978 *Parapuzosia* *seppenradensis* (Landois); Summesberger, p. 128, pl. 6, Fig 26; figs 16–17 (= *P.* (*P.*) *leptophylla*). [19]

1995 *Parapuzosia* (*Parapuzosia*) *seppenradensis* (Landois, 1895); Kennedy and Kaplan, p. 21, pls 35 – 41 (with additional synonymy on the Münster specimens) [20]

2005 *Parapuzosia* (*Parapuzosia*) *seppenradensis* (Landois, 1895); Kaplan, Kennedy and Hiß, p. 54, pl. 4. [21]

2006 *Parapuzosia* (*Parapuzosia*) *seppenradensis* (Landois, 1895); Kaplan, Kennedy and Scheer, p. 32, pls 3 – 6.[22]

2008 *Parapuzosia* (*P.*) *bosei* Scott and Moore; Kennedy, p. 157, Fig 20[in 23]

2016 *Parapuzosia* (*Parapuzosia*) *seppenradensis* (Landois, 1895); Kaplan, p. 52, figs 3, 4a–b, 5a (with additional synonymy on the Münster specimens). [24]

Types. Lectotype is the specimen figured by Landois [1, pl. 2], by subsequent designation of Kennedy [3, p. 21]. This unregistered specimen is exposed at the entrance of the LWL-Museum für Naturkunde, Münster, Germany. It was re-figured by Kennedy and Kaplan [3, pl. 35 – 37], by Kaplan [2, fig. 5b], and by many others. Paralectotype, housed in the permanent exposition of the LWL-Museum für Naturkunde, Münster, Germany, is the smaller specimen of Landois [1, pl. 1], re-figured by Kennedy and Kaplan [3, pls 38 – 39]. A precise reconstruction of the finding and levels of origin was presented by Kaplan [2].

Emended diagnosis. *Parapuzosia* (*Parapuzosia*) with five typical ontogenetic stages, in which the adult stage presents a broadly rounded venter and wide shallow ribs and reaches diameters (including the body chamber) of more than one meter in micro- and up to two meters in macroconchs.

Description. Large conch with a moderately compressed whorl section throughout all growth stages.

Growth stage 1 is characterized by sharp primary ribs and dense secondary ribs. The smallest individuals known from this species, up to a diameter of ca. 0.18 m, show about 9 primary ribs per half whorl, intercalated by six to nine secondary ribs. The discoidal shell is evolute to involute with a moderate expansion rate. The steep umbilical wall bends into the flank in a narrow bow, almost a ridge. Flanks converge, with greatest width at ca. one third of the whorl height. The venter is narrowly rounded. Straight, crescentic primaries arise near the umbilicus and become moderately prorsiradiate in a wide bow between mid-flank and the venter, which they cross. Seven to nine thin secondaries arise at mid-flank and parallel the primaries.

Growth stage 2 develops at D of 0.2 m to 0.4 m and is characterized by a decrease of secondaries from >6 to three per whorl. This ontogenetic stage resembles the juvenile stage 1 with respect to shell shape and primary ribbing.

Growth stage 3. Secondaries disappear between 0.2 m-0.4 m diameter; subsequent ornamentation thus solely consists of thin, straight primary ribs on the flanks. Primary ribs are dense and fine, flanks are convergent and the venter narrowly rounded. The shell is increasingly compressed and discoidal, but greatest whorl width is still at mid-flank. The venter appears to be smooth. This ontogenetic stage corresponds to smaller specimens identified as *P. americana* Scott and Moore, 1928 in North America*.*

Growth stage 4 is either smooth, or presents shallow, wide primaries, while the venter is rounded and widens slightly. This growth stage is developed at D ranging between 0.35-0.8 m. Where present, ribs become slightly broader, shallow and wide. Although the venter is wider rounded, flanks still converge and widest whorl width is still maintained at about one third of the whorl height. This ontogenetic stage corresponds to *P. americana* Scott and Moore, 1928*[16].*

Growth stage 5. Between 0.5 and 1.4 m, the venter gradually becomes even more widely rounded and whorls are widest at mid-flank. Flanks are approximately parallel. Primaries are broad and flat, and an undulating shell surface is developed. This ontogenetic stage corresponds to specimens identified as *P. bosei* Scott and Moore, 1928, but also the phragmocone of the lectotype of *P. seppenradensis* from Germany.

The suture is strongly incised, with L asymmetrically trifid, widely extended, and occasionally protruding into the previous suture (S1 Fig.).

Dimensions see Table S 1. Morphometry and distribution of *Parapuzosia* specimens studied here.

Discussion. The giant *Parapuzosia* of Tepeyac, Mexico, has been studied before. Böse[18] described three localities with giant *Parapuzosia* in the Jiménez area, the town closest to Tepeyac in northern Coahuila, Mexico. Their field notes were reproduced in more detail by Renz[19]. Young[20] visited this area and documented five small specimens (D ~0.45 m) as *P.* aff. *bradyi*, three small specimens (D 0.1 to 0.19 m) as *P. paulsoni* n.sp., and a single specimen (D ~0.45 m) as *P. terryi* n.sp. The author additionally assigned five small-sized specimens (D 0.11 to 0.19 m) to *P. bosei* Scott and Moore, 1928, including two incomplete individuals described previously by Renz[19]. as *P. corbarica*.

The German specimens of *P.* (*P.*) *seppenradensis* invariably have stronger ornament than the specimens from Mexico and England. Almost smooth specimens of *P.* (*P.*) *seppenradensis* are known from chalk or micritic carbonate sediment, whereas those presenting stronger ornament are majorly known from sandstone; this difference in shell morphology is therefore here interpreted to correspond to different hydrodynamic environments; a similarly wide range in shell ornament has been documented in *Schloenbachia varians* Sowerby, 1817, which developed a stronger ornament in high energy sedimentary environments and is smooth under low energy conditions [64]. This may also be the case here.

Size dimorphism was interpreted to be present in the German *Parapuzosia seppenradensis,* based on a specimen found in 1900 in the type area near Seppenrade[2], with convergent flanks and broad, shallow ribs. It is a juvenile of 800 mm shell diameter which is here referred to growth stage 4; it thus represents a macroconch.

Morphologies summarized here as ontogenetic stages 1 to 5 have originally been described as different species. North American species of giant *Parapuzosia* have always been regarded as closely related to each other[16, 19, 21], but ontogenetic stages or intraspecific variability have never been considered due to the availability of too few specimens.

*Parapuzosia americana* Scott and Moore, 1928 is a morphotype with convergent flanks and numerous primary ribs. The holotype is a specimen of 1.03 m diameter. The authors assumed that both whorl section and ornamentation would persist throughout ontogeny (e.g. in Pl. 38, p3), which is challenged by our material. Instead, we here interpret this specimen as a macroconch terminating in growth stage 4.

*Parapuzosia bosei* Scott and Moore, 1928, is based on a holotype of 0.95 m diameter. Its whorl section and ribbing correspond to *P.* (*P.*) *seppenradensis*, and it is here regarded to represent a microconch corresponding to growth stage 5.

*Parapuzosia paulsoni* Young, 1963 is characterized by sharp ribs, a broadly rounded venter and a simple suture, untypical of *Parapuzosia*. It is here excluded from this genus. The specimen was assigned to *Pachydiscus* by Cobban and Kennedy[65].

*Parapuzosia bradyi* Miller and Youngquist, 1946 is here considered to be a mature microconch of *P.* (*P.*) *seppenradensis*.

Juvenile stages 1 and 2 of *P.* (*P.*) *seppenradensis* are similar to *P.* (*P.*) *corbarica* (de Grossouvre, 1894)[66] from the Coniacian-Santonian of Spain, France, Austria and Poland. For this species, larger growth stages were never described. The strong morphological similarity was already noted [in 22] but but its relationship to the two giant species remains to be further elucidated.

Three growth stages of *P.* (*P.*) *daubreei* (de Grossouvre, 1894) are documented (compare specimens in [67] and [68], but the number of primary ribs throughout these ontogenetic stages is higher and they are generally coarser than in *P.* (*P.*) *seppenradensis*. *P.* (*P.*) *daubreei* clearly differs from all other *P.* (*Parapuzosia*).

Several large *Parapuzosia* from the upper Santonian – lower Campanian English Chalk and an undescribed specimen from the lower Santonian (?) of Southeast France[12] have been referred to *P.* (*P.*) *leptophylla* [13, e.g. 69, 70], based on irregular delicate ribbing and the cross sections of these secimens. The holotype of *P.* (*P.*) *leptophylla* [6, pl. 22, figs 1a-b] from the Upper Chalk of Kent has convergent flanks, a narrowly rounded venter and 34 delicate ribs per whorl in the juvenile stage. The type of ribbing and the invariably smooth adult stage separate *P.* (*P.*) *leptophylla* from (*P.*(*P.*) *seppenradensis*, as already discussed[7]. Morphometrically, *P.* (*P.*) *leptophylla* is indistinguishable from *P.* (*P.*) *seppenradensis* (S3 Fig). Rather, differences exist regarding adult diameter and ornamentation during ontogenesis: *P.* (*P.*) *leptophylla* never develops an adult stage with broad, shallow ribs, parallel flanks and a broadly rounded venter, but instead, its shell terminates at stages corresponding to growth stage 4 of *P.* (*P.*) *seppenradensis*. In addition, ribbing is irregular in *P.* (*P.*) *leptophylla* in earlier growth stages but is very regular in growth stage 3 of *P.* (*P.*) *seppenradensis,* or shells are smooth. Earliest growth stages are unknown from *P.* (*P.*) *leptophylla*.

Occurrence. Latest Santonian of the north-western Gulf of Mexico and England, early Campanian of Germany, north-western Gulf of Mexico and England. The age of the specimen from Montana is unknown. Specimens >> 1 m are exclusively early Campanian in age.

# Additional references cited

54. Wright CW, Calloman [sic] JH, Howarth MK. Cretaceous Ammonoidea. In: Kaesler RL, editor. Treatise on Invertebrate Paleontology. Part L, Mollusca 4, revised. 2nd ed. Boulder, Lawrence: University of Kansas & Geological Society of America; 1996. p. xx+362.

55. Cuvier G. Tableau élémentaire de l'histoire naturelle des Animaux. Paris: Baudouin; 1797. xvi+710 p.

56. von Zittel KA. Handbuch der Palæontologie 1. Abtheilung. Palæozoologie 2. Band. Mollusca und Arthropoda. Schimper WP, Schenk A, Scudder SH, editors. München: R. Oldenbourg; 1884. 893 p.

57. Hyatt A. Genesis Of The Arietidae. Washington, DC1889. xi+238 p.

58. von Zittel KA. Grundzüge der Paläontologie. München, Germany: R. Oldenbourg; 1895. viii+971 p.

59. Spath LF. On the Senonian ammonite fauna of Pondoland. Transactions of the Royal Society of South Africa. 1922;10:113-48.

60. Nowak J. Untersuchungen über die Cephalopoden der oberen Kreide in Polen III. Teil. Bulletin international de l’Académie des Sciences de Cracovie, Classe des Sciences mathématiques et naturelles, Série B, Sciences naturelles. 1913;1913:335-415.

61. Fraas O. Riesenammoniten. Jb Verein vaterlandl Naturk Württemb. 1891;47:441-2.

62. Schlüter C. *Podocrates* im Senon von Braunschweig und Verbreitung und Benennung der Gattung. Zeitschr Dt Geol Ges. 1899;51(3):409-30.

63. Köplitz W. Über die Fauna des oberen Untersenon im Seppenrade-Dülmener Höhenzuge [Dissertation]. Münster: Westfälische Wilhelms-Universität; 1920.

64. Wilmsen M, Mosavinia A. Phenotypic plasticity and taxonomy of *Schloenbachia varians* (J. Sowerby, 1817) (Cretaceous Ammonoidea). Paläontologische Zeitschrift. 2011;85:169-84. doi: 10.1007/s12542-010-0086-5;

65. Cobban WA, Kennedy WJ. Campanian *Trachyscaphites spiniger* ammonite fauna in north-east Texas. Palaeontology. 1992;35(1):63-93.

66. de Grossouvre A. Recherches sur la Craie Supérieure, 2, Paléontologie. Les ammonites de la Craie Supérieure. Paris: Imprimerie Nationale; 1894. ii+264 p.

67. Remin Z. Upper Coniacian, Santonian, and lowermost Campanian ammonites of the Lipnik-Kije section, central Poland—taxonomy, stratigraphy, and palaeogeographic significance. Cretaceous Research. 2010;31:154-80.

68. Kennedy WJ, Bilotte M, Melchior P. Ammonite faunas, biostratigraphy and sequence stratigraphy of the Coniacian-Santonian of the Corbières (NE Pyrénées). Bulletin des Centres de Recherches Exploration-Production Elf-Aquitaine. 1995;19(2):377-499.

69. Bailey HW, Gale AS, Mortimore RN, Swiecicki A, Wood CJ. Biostratigraphical criteria for the recognition of the Coniacian to Maastrichtian stage boundaries in the Chalk of north-west Europe, with particular reference to southern England. Bulletin of the Geological Society of Denmark. 1984;33:31-9.

70. Gale A, Montgomery P, Kennedy W, Hancock J, Burnett J, McArthur J. Definition and global correlation of the Santonian‐Campanian boundary. Terra Nova. 1995;7(6):611-22.

1. Cuvier G. Tableau élémentaire de l'histoire naturelle des Animaux. Paris: Baudouin; 1797. xvi+710 p.

2. von Zittel KA. Handbuch der Palæontologie 1. Abtheilung. Palæozoologie 2. Band. Mollusca und Arthropoda. Schimper WP, Schenk A, Scudder SH, editors. München: R. Oldenbourg; 1884. 893 p.

3. Hyatt A. Genesis Of The Arietidae. Washington, DC1889. xi+238 p.

4. von Zittel KA. Grundzüge der Paläontologie. München, Germany: R. Oldenbourg; 1895. viii+971 p.

5. Spath LF. On the Senonian ammonite fauna of Pondoland. Transactions of the Royal Society of South Africa. 1922;10:113-48.

6. Nowak J. Untersuchungen über die Cephalopoden der oberen Kreide in Polen III. Teil. Bulletin international de l’Académie des Sciences de Cracovie, Classe des Sciences mathématiques et naturelles, Série B, Sciences naturelles. 1913;1913:335-415.

7. Landois H. Ueber einen ungewöhnlich grossen Ammonites Coesfeldiensis Schl. Zeitschr Dt Geol Ges. 1887;39(3):612-3.

8. Fraas O. Riesenammoniten. Jb Verein vaterlandl Naturk Württemb. 1891;47:441-2.

9. Landois H. Die Riesenammoniten von Seppenrade: Pachydiscus ZITTEL Seppenradensis H. LANDOIS. Jahresbericht des Westfälischen Provinzialvereins für Wissenschaft und Kunst. 1895;23:99-108.

10. Schlüter C. *Podocrates* im Senon von Braunschweig und Verbreitung und Benennung der Gattung. Zeitschr Dt Geol Ges. 1899;51(3):409-30.

11. Köplitz W. Über die Fauna des oberen Untersenon im Seppenrade-Dülmener Höhenzuge [Dissertation]. Münster: Westfälische Wilhelms-Universität; 1920.

12. Böse E, Cavins OA. The Cretaceous and Tertiary of southern Texas and northern Mexico. Univ Texas Bull. 1927;2748:7-142.

13. Scott G, Moore MH. Ammonites of enormous size from the Texas Cretaceous. J Paleont. 1928;2(4):273-8.

14. Adkins WS. Handbook of Cretaceous fossils. Univ Texas Bull. 1928;2838:1-303.

15. Böse E. Cretaceous ammonites from Texas and northern Mexico. Univ Texas Bull. 1928;2748:143-357.

16. Renz HH. Neue Cephalopoden aus der oberen Kreide von Rio Grande del Norte (Mexico und Texas). Abhandlungen der Schweizerischen Paläontoloogischen Gesellschaft. 1936;57:1-16.

17. Miller AK, Youngquist W. A giant ammonite from the Cretaceous of Montana. J Paleont. 1946;20(5):479-84.

18. Young K. Upper Cretaceous ammonites from the Gulf Coast of the United States. Univ Texas Bull. 1963;6304:1-373.

19. Summesberger H. Eine obersantone Ammonitenfauna aus dem Becken von Gosau (Oberösterreich). Annalen des Naturhistorischen Museums in Wien. 1978:109-76.

20. Kennedy WJ, Kaplan U. *Parapuzosia* (*Parapuzosia*) *seppenradensis* (Landois) und die Ammonitenfauna der Dülmener Schichten, unteres Unter-Campan, Westfalen. Geologie und Paläontologie in Westfalen. 1995;33:127 pp.

21. Kaplan U, Kennedy WJ, Hiß M. Stratigraphie und Ammonitenfaunen des Campan im nordwestlichen und zentralen Münsterland. Geologie und Paläontologie in Westfalen. 2005;64:1-171.

22. Kaplan U, Kennedy WJ, Scheer U. Ammoniten der Bottrop-Formation, Campanium, westliches Münsterland. Geologie und Paläontologie in Westfalen. 2006;67:1-71.

23. Gale AS, Hancock JM, Kennedy WJ, Petrizzo MR, Lees JA, Walaszczyk I, et al. An integrated study (geochemistry, stable oxygen and carbon isotopes, nannofossils, planktonic foraminifera, inoceramid bivalves, ammonites and crinoids) of the Waxahachie Dam Spillway section, north Texas: a possible boundary stratotype for the base of the Campanian stage. Cretaceous Research. 2008;29:131-67. doi: 10.1016/j.cretres.2007.04.006;

24. Kaplan U. Ein neues Exemplar von *Parapuzosia* (*Parapuzosia*) *seppenradensis* (Landois, 1895) aus dem Typusgebiet von Seppenrade, Münsterland. Geologie und Paläontologie in Westfalen. 2016;88:49-61.
