# Supplementary material for: Ontogeny, evolution and palaeogeographic distribution of the world’s largest ammonite Parapuzosia (P.) seppenradensis (Landois, 1895)
Source: PLoS One. 2021 Nov 10;16(11):e0258510. doi: 10.1371/journal.pone.0258510 (PMC8580234; doi:10.1371/journal.pone.0258510)
Supplement: S2 File — (DOCX) [file pone.0258510.s009.docx]

# S5 file

# Details on the Tepeyac locality

Böse[18] described three localities with giant *Parapuzosia* in the Jiménez area in northeastern Mexico: Arroyo Tecolote, Arroyo Blanco and Arroyo El Fresno. His field notes were reproduced in more detail by Renz}[19]. Young[20]also visited this area. From the data of these authors, we infer that the Tepeyac locality comprises the Arroyo Blanco and Arroyo Tecolote localities of Böse[18].

A kmz-file with the position of each specimen is available from CI upon request.

# Additional references cited

73. Servicio Geológico de México. Carta Geológica Minera 1 : 250 000, H14-7 and H14-10. 6th ed. México D.F., México2008.

74. Salvador A. Origin and developmant of the Gulf of Mexico Basin. In: Salvador A, editor. The Gulf of Mexico Basin. The Geology of North America. J. Geological Society of America, Boulder, Colorado1991. p. 389-444.

75. Sohl NF, Martinez RE, Salmerón-Ureña P, Soto-Jaramillo F. Upper Cretaceous. In: Salvador A, editor. The Gulf of Mexico Basin. The Geology of North America. J. Geological Society of America, Boulder, Colorado1991. p. 205-215
